# Behavioral relevance of category selectivity revealed by human ECoG data

**DOI:** 10.1371/journal.pone.0328374

**Published:** 2025-10-07

**Authors:** Seyedamin Hashemi, Seyed Ali Ebadi, Fatemeh Zareayan Jahromy, Stefan Treue, Gabriel Kreiman, Moein Esghaei

**Affiliations:** 1 School of Electrical, Computer and Energy Engineering, Arizona State University, Tempe, Arizona, United States of America; 2 Department of electrical engineering, Amirkabir University of Technology (Tehran Polytechnic-AUT), Tehran, Iran; 3 Department of Biomedical Engineering, School of Electrical Engineering, Iran University of Science and Technology (IUST), Tehran, Iran; 4 Neuroscience Laboratory, German Primate Center - Leibniz Institute for Primate Research, Goettingen, Germany; 5 Faculty of Biology and Psychology, University of Göttingen, Göttingen, Germany,; 6 Harvard Medical School, Harvard University, Cambridge, Massachusetts, United States of America,; 7 Faculty of Computer Science and Engineering, Shahid Beheshti University, Tehran, Iran; Kochi University of Technology, JAPAN

## Abstract

Object recognition is a crucial brain function that involves a complex interplay between various brain regions. However, the behavioral relevance of functional interactions between these regions remains largely unexplored. In this study, we examined the functional interactions between different brain regions during object recognition using intracranial electrocorticography (ECoG) recordings in subjects diagnosed with pharmacologically intractable epilepsy. We computed the phase locking value (PLV) between different brain areas and its category selectivity, and assessed its behavioral relevance by comparing correctly and incorrectly performed trials. Our results revealed that phase locking between brain regions varies across different object categories and that this variability significantly influences the perceptual behavior of subjects. Importantly, we found that the behavioral relevance of these interactions is spatially organized, with the high behaviorally relevant connections being longer for the frontal lobe and shorter for the occipital lobe. These findings underscore the unique roles of different brain areas in object recognition and pave the way for more nuanced explorations of the interplay between brain regions in object recognition and other cognitive functions.

## Introduction

Understanding object categories is vital for brain functionality, explored via neural recordings and functional neuroimaging [1–5]. Research emphasizes the ventral visual pathway, especially the IT cortex’s role in object identification [1–6]. However, there is debate over whether the temporal area’s neural information conveys semantic category information or is purely influenced by object shape [7–11].

A simplified explanation for the development of category preferences from shape-selective responses suggests that visual features differentiating object categories, likely represented in the IT cortex, aid in classifying objects into semantic groups by classifiers in areas like the prefrontal cortex (PFC). The PFC is believed to integrate visual information with memory for task-relevant decisions and send modulatory feedback to visual areas [8,12]. Aligning with this idea, there are proposed object recognition models that construct detailed object representations through hierarchical layering of linear and non-linear processes, assembling a shape feature lexicon that a classifier can decode into categorical labels [13–16]. Studies show IT cortex‘s category-selective activity is mostly shape-based, while the PFC exhibits flexible, task-dependent activity aiding categorization [8,17–20].

Object recognition likely encompasses a broad spectrum of sensory and higher order cognitive functions. Early visual areas handle the initial processing of visual stimuli, while higher cognitive tasks like conscious perception, learning, and decision-making occur in upper cortical areas, such as the frontal cortex. Despite the physical distance between neural systems for these functions [2,3,21], they all depend on inputs from early visual areas. The nature of the interactions among brain regions responsible for various levels of processing during object recognition remains largely unknown. Here, we specifically examine the critical nature of functional interactions between brain regions for the identification of object categories, and explore the extent to which the efficacy of these interactions in category identification is influenced by their spatial arrangement.

Previous studies have computed the strength of phase locking between activities of different brain regions, as proxy of neural communication between them [22–26]; however, it is not yet clear how functionally as well as behaviorally relevant this coupling may be for identification of object categories in human brain (see however [27] for a documentation of content-specific frontal-parietal phase coupling in monkeys). In order to examine these interactions, we recorded electrocorticography (ECoG) field potentials from epilepsy patients and measured the strength of phase locking between different brain areas [28]. We further investigated if this inter-areal synchrony is informative of different object categories presented to the human subjects. Our data show not only that the inter-areal synchrony pattern is category-dependent but that the spatial pattern of this category selectivity is behaviorally relevant, i.e., the highest behavioral relevance of category selectivity was evident in shorter connection for the occipital lobe and longer connections for the frontal areas. These results highlight the anatomical dependence of the contribution of shorter neuronal connections vs. longer connections to the transmission of object category information in the human brain.

## Materials and methods

### Experimental procedure

#### Participants.

Participants were 10 people, aged between 12–34, diagnosed with pharmacologically intractable epilepsy. The recordings were performed in either Children’s Hospital Boston (CHB) or Brigham and Women’s Hospital (BWH), with the aim of localizing the seizure foci for potential surgical resection. For more detailed information on the dataset, see the original publication on the data [28] and the license agreement in Kreiman Lab (https://klab.tch.harvard.edu/code/code.html).

### Ethics statement

All participants provided informed consent and were ensured about the confidentiality of their data. The study was approved by the institutional review boards at both Children’s Hospital Boston (CHB) and Brigham and Women’s Hospital (BWH).

### Recordings and preprocessing

Intracranially implanted electrodes (Ad-Tech, Racine, WI, USA; each recording site was 2 mm in diameter with 1 cm separation) [29–30] were utilized to localize the seizure event foci. Recording regions were set out in grids or strips, each containing 4–64 recording sites.). The number of electrodes per participant ranged from 48 to 126 (mean ± SD: 80 ± 18). As a result, the number of electrode pairs varied across individuals, from approximately 1,128 (for 48 electrodes) up to 7,875 (for 126 electrodes). On average, each participant had around 3,160 pairs. All recorded neural signals were amplified (×2500), filtered between 0.1 and 100 Hz, and sampled with 256 Hz sampling rate at CHB (XLTEK, Oakville, ON, Canada) and 500 Hz at BWH (Bio-Logic, Knoxville, TN, USA). The data were further denoised using a 60 Hz notch filter and each electrode’s signal was globally referenced [28]. Although this referencing approach may suffer from global artifacts, it maintains neuronal activity relevant to the macroscopic (rather than mesoscopic) scale of brain state (see [31] for a mesoscopic approach). Subjects were hospitalized 6–9 days, during which physiological data were continuously monitored. The data presented in this study is based on the periods without seizure activity.

### Stimulus presentation

Objects from five different categories (five different examples per category) were presented as contrast-normalized grayscale images to subjects: “animals,” “chairs,” “human faces,” “fruits,” and “vehicles.” Contrast normalization was done by fixing the grayscale pixel level standard deviation. Images were shown for 200 ms separated by an interval of 600 ms, while subjects performed a one-back memory task to detect the repeated presentation of the same image category. A pseudorandom order was used to present the images. The details of the task and the images are provided in the supplementary materials of reference [28].

### Data analysis

#### Phase locking value (PLV).

Recorded data, spanning from target stimulus onset to completion (900 ms), were filtered between 4–32 Hz in 4 Hz-wide bands with an overlap of 2 Hz using the EEGLAB toolbox [32] in MATLAB (The MathWorks, Natick, MA). For each frequency band, we calculated the PLV between the recorded signals from each pair of electrodes: A unit-length vector was assigned to each temporal sample (i.e., a discrete time point within the recorded data) with its phase equal to the phase difference between the two electrodes. These vectors were averaged over temporal samples, and finally the length of resultant vectors was averaged over trials (Equation 1):


$$PLV=\;\frac{1}{M}\sum_{h=1}^{M}\left|\frac{1}{N}\sum_{k=1}^{N}e^{j|\varphi_{1}(h,k)-\varphi_{2}(h,k)|}\right|$$ (1)

Where h indexes the trials, with the total number of M; k indexes the temporal samples within each trial, with the total number of N; and ϕ_1_ and ϕ_2_ are the instantaneous phase for the signals recorded from electrodes 1 & 2, derived using the Hilbert transform and the term j equals $\sqrt{-1}$. PLV represents the magnitude of phase locking between the given electrode pair.

#### Category selectivity (RV).

To calculate the category selectivity of PLV, the across-category variation of PLV was divided by the average within-category variation of PLV (a measure called RV):


$$RV=\;\frac{\sigma (\mu \left({PLV}_{cat1}\right),\mu \left({PLV}_{cat2}\right),\mu \left({PLV}_{cat3}\right),\mu \left({PLV}_{cat4}\right),\mu ({PLV}_{cat5}))}{\mu (\sigma \left({PLV}_{cat1}\right),\sigma \left({PLV}_{cat2}\right),\sigma \left({PLV}_{cat3}\right),\sigma \left({PLV}_{cat4}\right),\sigma \left({PLV}_{cat5}\right))}$$ (2)

Where σ represents the standard deviation, μ denotes the average and cat_i_ stands for the *i*th category. Higher values of RV reflect a larger distinction between the different categories’ PLVs.

#### Behavioral relevance of category selectivity.

To compute how different category selectivity is between hit and miss trials, for each category, RV was computed for a randomly selected subset of hit trials with the same size as the miss trials. This procedure was repeated for 50 iterations and the z-score of RVs for miss vs. hit trials was calculated:


$$\text{ Behavioral relevance }=\;\frac{\mu \left({RV}_{hit}\right)\;-\;{RV}_{miss}}{\sigma \left({RV}_{hit}\right)}$$ (3)

where RV_miss_ and RV_hit_ are computed over the set of miss trials and a given subset of hit trials, respectively and μ/σ are calculated over the randomly selected subsets of hit trials (with an equal size as the miss trials). This measure reflects how strongly category selectivity is linked to the subject’s behavioral performance.

The dependence of behavioral relevance on the distance between the areas was examined for each region by comparing the lateral length of the connections from the lowest and highest octiles of behavioral relevance. To this end, the absolute difference in lateral length between the highest and lowest behavioral relevance octiles was computed. To quantify the significance of this difference, a random selection of connections with the same size as the high and low behaviorally relevant groups were made for each area (for 100,000 repetitions) and the difference in lateral length was computed for each repetition. Next the original absolute difference was compared to this distribution of the randomly generated data, resulting in a p-value by calculating the proportion of random differences that are less likely than the original difference value.

## Results

**Fig 1:**
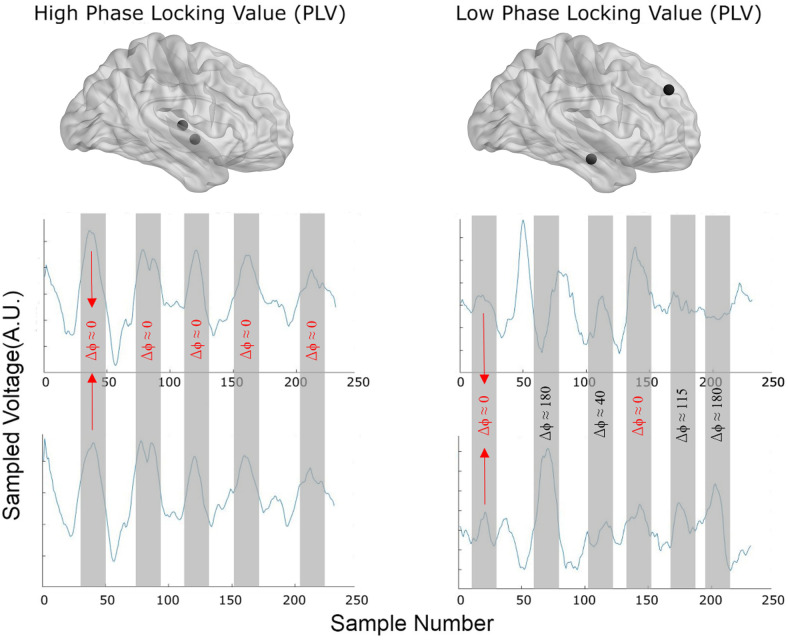
Example signals for different PLV magnitudes. Sample IFP signals recorded from two different pairs of electrodes (256 Hz sampling rate) with low (0.34) and high (0.90) Phase Locking Value (PLV). As highlighted, the peaks and troughs from the two recording electrodes are well aligned in the high PLV condition (left column), whereas no clear alignment is observed in the low phase locking condition (right column). Electrodes come from the middle temporal gyrus and superior lateral temporal gyrus (left) and medial Parahippocampal gyrus and middle frontal gyrus (right).

We examined whether the functional interaction between two different brain regions has any role in the encoding of distinct visual object categories. To investigate this, we recorded Electrocorticogram (ECoG) signals from different brain regions of epileptic patients, while they performed a one-back memory task involving different object categories. We calculated the degree of phase-locking between the intracranial field potentials (IFPs) of each pair of electrodes by measuring the phase locking value (PLV) between the recorded signals for each of the different frequency bands ranging between 4–32 Hz (see Methods – Equation 1). Fig 1 shows example data from two different pairs of electrodes with high, and low PLVs (0.92 and 0.36, respectively). The location of each electrode pair is shown in the brain insets. The left panel representing a high PLV depicts how the phase difference between the two electrodes remains constant across time, whereas the two signals in the right panel do not maintain a fixed phase difference through time. This is an example of how variable the inter-areal functional interaction could be, motivating the question whether this variation contributes to the processing of object categories.

**Fig 2:**
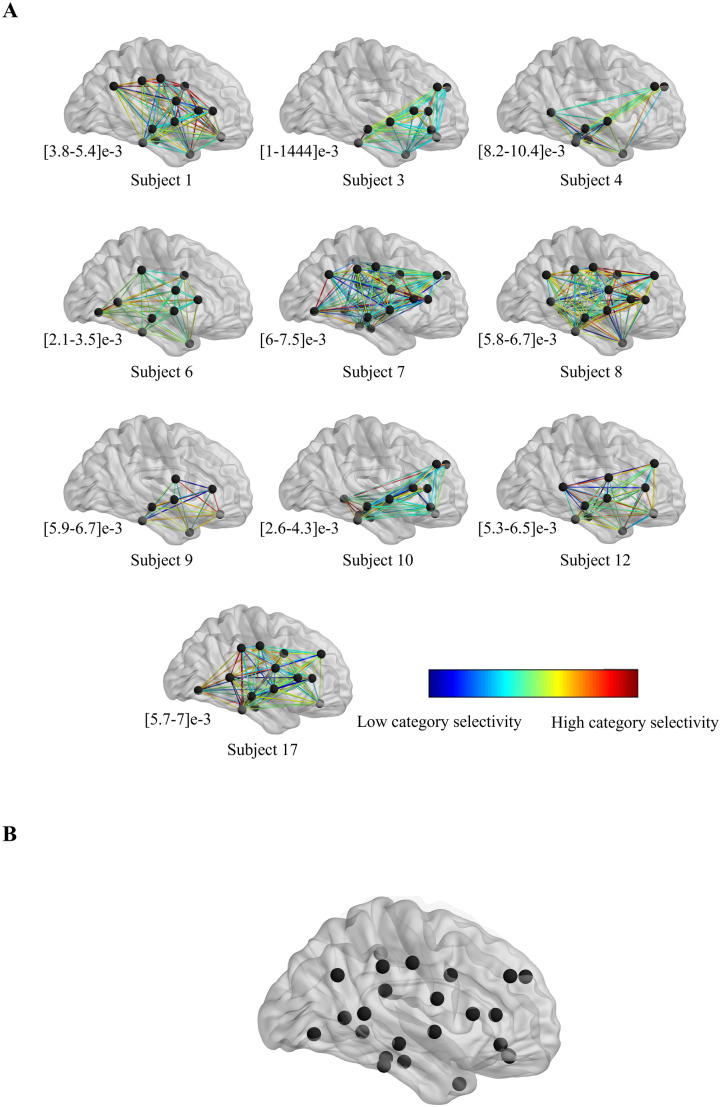
PLV-based category selectivity for different pairs of brain regions. **(A)** Brain maps show the location of regions at which at least one electrode was placed for each subject and the strength of PLV-based category selectivity for each pair of brain regions. The numbers beneath each subject indicate the range of PLV-based category selectivity for that subject. **(B)** Location of analyzed brain regions (those regions that have been recorded in more than one subject [N = 24]).

We next computed (for each frequency band) the category selectivity of each electrode pair’s PLV, by dividing the variation (across categories) of PLV by the mean of PLV variation within each category (see Methods – Equation 2. To enable across-subject analysis of the data, we focused our analyses on only those regions which were recorded in at least two different subjects and averaged this category selectivity measure across the low frequency bands. We next computed the category selectivity for the PLV between all possible pairs of electrodes within each subject’s data. For each pair of brain regions then the category selectivity was averaged between the corresponding electrode pairs (Fig 2A). The strength of category selectivity for each pair of regions is color-coded, showing its variability within and across subjects. The analyzed regions (n = 24) across the dataset are shown in the across-subject super map (Fig 2B). Observing the map of PLV-based category selectivity for individual subjects clearly visualizes how category selectivity varies across different pairs of brain regions. These 24 regions are determined based on clinical criterion which is explained in detail in [28].

**Fig 3:**
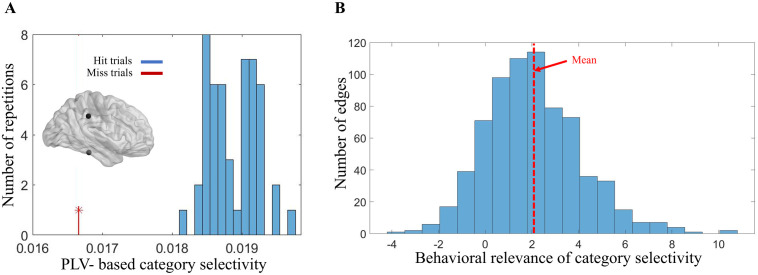
Distribution of category selectivity and its behavioral relevance for pairs of brain regions. (A) An example edge’s behavioral relevance (z-score = 6.4) for a sample subject; Each of the histogram’s data points represents the category selectivity (calculated using Eq. 2) for one randomly selected subset of hit trials (with the same number as the miss trials). The data point shown with a red line indicates the category selectivity computed for the miss trials (N = 309) of the sample subject (subject 6). Obviously, the miss trials’ category selectivity is considerably separated from that of the hit trials selected subsets. (B) Histogram of category selectivity’s behavioral relevance (Eq. 3) across significant and non-significant edges of all subjects with each data point representing the behavioral relevance for each of 715 edges (p < 1e-78, sign test).

In the final step, we computed the behavioral relevance of RV, by randomly selecting a subset of hit trials equal to the number of wrongly performed (miss) trials in each category (132.6 ± 85.44 miss trials across all subjects) for 50 repetitions and calculating the z-score of RV for miss relative to hit trials (see Methods) (Fig 3A). Fig 3B shows the distribution of behavioral relevance across all possible edges (inter-regional connections) of all subjects which is significantly above zero (p < 1e-78, sign test), indicating that category selectivity of inter-regional functional interaction needs to be large enough for the subjects to accomplish the trial successfully. These results suggest that not only the functional interaction between regions (reflected by PLV) is different across the presentation of different object categories, but also that this difference is relevant for the perceptual behavior of the subjects.

**Fig 4:**
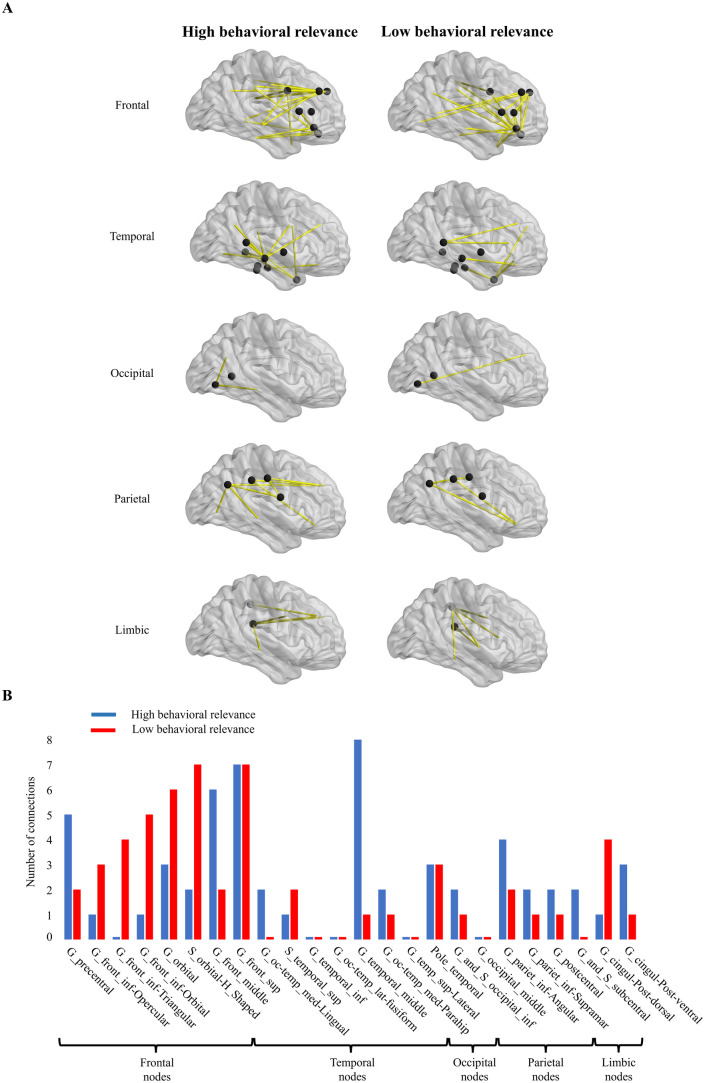
Different brain regions’ contribution to the behavioral dependence of connectivity. (A) Left and right columns represent connections from the highest and lowest quantile (octile), respectively of behavioral relevance, separated by involvement of each brain lobe. (B) Distribution of connections for each region, categorized by behavioral relevance, with high behavioral relevance nodes in blue and low behavioral relevance nodes in red.

**Fig 5:**
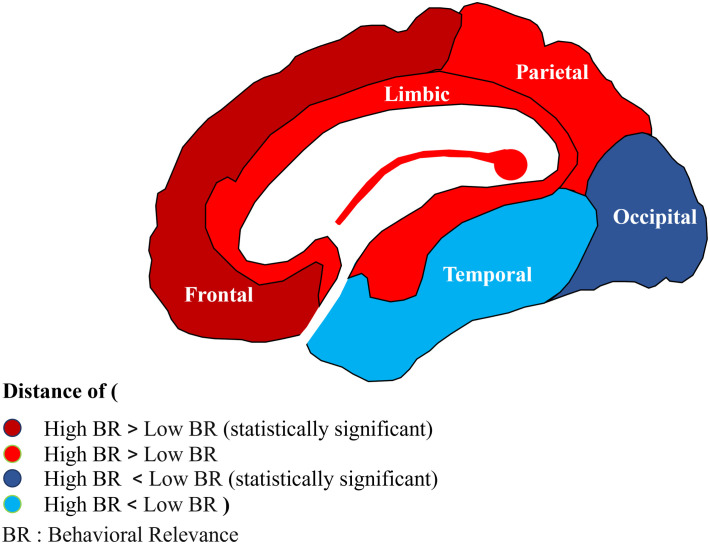
Behavioral relevance of category selectivity for each brain lobe. Lighter-colored regions are the regions with no significant p-values (parietal, temporal, and limbic), red indicates the regions with a longer distance for connections with stronger behavioral relevance, and blue in contrast shows a shorter distance for connections with stronger behavioral relevance.

To investigate the spatial organization of behaviorally relevant edges across the brain, we averaged each edge’s behavioral relevance across all subjects and consequently partitioned them into eight groups sorted by their behavioral relevance. We next selected the groups with the lowest and highest behavioral relevance. Fig 4A shows the two groups of edges categorized into the main anatomical brain lobes (“frontal,” “parietal,” “temporal,” “limbic” and occipital) based on either of their ending points. Fig 4B quantifies the contribution of each region to the high/low behavioral relevance network, by counting the number of edges from each region separated by behavioral relevance. For the frontal lobe it is obvious that the edges with the highest behavioral relevance are mostly those which connect distant brain areas, whereas the connections with the lowest behavioral relevance are shorter (p-value = 0.03, permutation test). This effect is even more pronounced for electrodes in the prefrontal cortex (p-value = 0.009, permutation test). However, the opposite effect is observed for occipital (p-value = 0.006, permutation test) lobe (Fig 5). This is consistent with the proposal that the functional interaction of purely sensory areas (like occipital cortex) with closer neighboring areas is of more behaviorally crucial importance compared to the interaction with higher order areas. On the other hand, for a high-level associative area and particularly the prefrontal cortex (with a profound role in multi-modal processing), functional interaction (in terms of visual information) with the network of distantly located areas is of more behavioral relevance rather that interaction with anatomically close-by brain areas. A similar effect to that observed in the frontal lobe was also found in the parietal and limbic lobes, while the effect observed in the occipital lobe was likewise present in the temporal lobe. However, statistical analysis did not yield significant results for these lobes.

## Discussion

The role of global inter-areal neuronal interactions vs. localized intra-areal processing in representation and conscious perception of object information has been widely emphasized but its behavioral relevance remains unclear [33]. Our data show that i) there is a phase locking between electrode (region) pairs, ii) the behavioral performances are linked to the category selectivity of the phase locking value, and iii) the information about the behavior is dependent on the length of the connections between the areas. Specifically, for the frontal lobe, higher behavioral relevant connections tend to be longer however for the occipital lobe these connections tend to be shorter.

Normally, brain imaging studies on humans use imaging methods, such as fMRI, EEG, MEG, all suffering from a lack of either temporal resolution (as for fMRI) or spatial resolution (as for EEG and MEG). These limitations have been an obstacle for examining the fine time-resolved interaction of distant local brain regions. In this study, using ECoG electrodes, we recorded intracranially from a multitude of brain regions many reported to be involved in object categorization. This allowed us for the first time to address the behavioral relevance of inter-areal interactions for the representation of object categories. Our results are the first evidence for a potential role of inter-areal interaction in encoding of environmental object categories in the human brain, supporting the thesis that not only the single regions matter in object perception, but also their interaction plays an independent role in perceptual coding. While here we focused on the role of connectivity and the importance of region-dependent connection length in object recognition, understanding the directionality of the interactions may help us to better understand the causal role of regional nodes on neighboring regions.

Previous studies evaluated the role of information transfer and connections between different sensory and executive areas in the process of object recognition. In an experimental study [34] tested a model that could describe the full path of recognizing the identity of an object in the brain using fMRI data. In this model after early analysis, information about low spatial frequency components of a picture is transferred in a fast pathway from primary visual areas in cerebral cortex to frontal cortex which is sent back to temporal cortex to be combined with other information for final decision [34].

Our finding of the distinguished behavioral role of frontal lobe’s connectivity with distantly located regions is also consistent with previous observations where an impairment of long frontal-temporal pathways severely affected object discrimination performance [35–36]. Our result is further in-line with the prefrontal cortex’ unique setting, transmitting information to/from many distinct brain circuits and a critical role in a wide set of other critical cognitive abilities; attention, memory, decision making and perceptual binding [37–40]. Our observation aligns well with the existing evidence that emphasizes the importance of distributed neural networks for visual processing elucidating the distinct roles of frontal and occipital lobes in object recognition. The finding that higher behavioral relevant connections involving the frontal lobe are longer while such connections are shorter for occipital lobe, is consistent with the hierarchical organization of the visual system.

Although we found that the length of connections of higher and lower behavioral relevance connections varies across different brain regions, we did not observe a whole-brain relationship between connection length and behavior. Specifically, there was no significant length dependence of behavioral relevance for connections with the limbic, temporal, and parietal lobes.

While we chose a broad frequency window (4–32 Hz) to capture an overarching perspective of inter-areal connectivity in object coding, it would be informative in future work to parse this range into narrower, well-defined sub-bands. Such an analysis could further elucidate the distinct oscillatory mechanisms contributing to category representation and behavioral outcomes.

In conclusion, our study has advanced our understanding of object recognition by illustrating the critical role of functional interactions between distinct brain regions. It was demonstrated that the category selectivity of these interactions, reflected by phase-locking values, not only varies across different object categories but also significantly influences the perceptual behavior of subjects. Moreover, we found that the behavioral relevance of these interactions is spatially organized in a way that underscores the unique roles of different brain areas in object recognition. Specifically, connections exhibiting a high behavioral relevance for the frontal lobe are longer, while for the occipital lobe, these connections are shorter. These insights align with our understanding of the occipital cortex’s role in sensory processing and the prefrontal cortex’s role in multi-modal processing. Overall, these findings pave the way for more nuanced explorations of brain region interactions and their influence on object recognition and other cognitive functions
